# Using Wearable Sensors to Measure and Predict Personal Circadian Lighting Exposure in Nursing Home Residents: Model Development and Validation

**DOI:** 10.2196/72338

**Published:** 2025-09-11

**Authors:** Shevvaa Beiglary, Yanxiao Feng, Nan Wang, Neda Ghaeili, Ying-Ling Jao, Yo-Jen Liao, Yuxin Li, Julian Wang

**Affiliations:** 1 Department of Architectural Engineering, Pennsylvania State University, 556 White Course Drive, University Park, PA, 16802, United States, 1 814-865-6394; 2 School of Applied Engineering and Technology, New Jersey Institute of Technology, Newark, NJ, United States; 3 Department of Civil and Environmental Engineering, Northwestern University, Evanston, IL, United States; 4 Ross and Carol Nese College of Nursing, Pennsylvania State University, University Park, PA, United States

**Keywords:** circadian, lighting, wearable sensors, long-term care facilities, predictive modeling, personalized lighting assessment, dementia, Alzheimer disease

## Abstract

**Background:**

Lighting, especially circadian lighting, significantly affects people with dementia, influencing sleep patterns, daytime alertness, and behavioral symptoms such as agitation. Since individuals experience and respond to light differently, measuring personal lighting exposure is essential for understanding its impact on health. Without individual data, the connection between lighting and health outcomes remains unclear. Wearable sensors provide a practical way to track personal light exposure, helping researchers better assess its effects on circadian rhythms and overall well-being.

**Objective:**

This study aims to develop and validate both calibration and predictive models using wearable lighting sensors to assess individual circadian lighting exposure accurately. By leveraging machine learning techniques and empirical data, we seek to establish a reliable method for health care researchers and practitioners to investigate and optimize lighting conditions for improved circadian health in nursing homes, especially for residents with dementia.

**Methods:**

A combination of controlled laboratory experiments and on-site data collection was conducted using professional spectrophotometer measurements as ground truth. Calibration models were developed for photopic lux and correlated color temperature, while predictive models estimated circadian metrics such as circadian stimulus. The sensors and the developed models were implemented in a real-world health care research project about bright light therapy intervention at 2 assisted-living facilities.

**Results:**

The calibration models for photopic lux and correlated color temperature demonstrated strong accuracy, with an adjusted *R*² of 0.858 and 0.982, respectively, ensuring reliable sensor measurements. Predictive models for circadian stimulus were developed using both simple regression and machine learning techniques. The random forest model outperformed linear regression, achieving an adjusted *R*² of 0.915 and a cross-validation *R*² of 0.857, demonstrating high generalization capability. Upon the implementation of these models, significant individual variations in circadian light exposure were found in the study, highlighting the significance of customized lighting evaluations. These results confirm the effectiveness of wearable sensors, combined with the developed calibration and predictive modeling, in accurately assessing personal circadian light exposure and supporting lighting-related health care research.

**Conclusions:**

This study introduces an effective and scalable approach to circadian light assessment using wearable sensors and predictive modeling. By replacing labor-intensive and costly spectrometer measurements, the proposed methodology enables continuous, cost-effective monitoring in health care environments. However, challenges related to sensor wearability, durability, and user compliance were identified, underscoring the need for further sensor design refinements. Future research should focus on refining sensor integration, expanding case studies, and developing adaptive lighting interventions to enhance circadian health in vulnerable populations.

## Introduction

### Background of Circadian Lighting and Health

The human circadian system is a complex biological mechanism that regulates various physiological and behavioral processes, including sleep-wake cycles, hormone secretion, and cognitive function [1]. This system is primarily entrained by light-dark cycles, with exposure to light during the day and darkness at night being crucial for maintaining a healthy circadian rhythm [2,3]. Disruption of the circadian system, commonly known as circadian rhythm disruption, has been associated with a wide range of adverse health outcomes, including sleep disorders, metabolic disorders, cardiovascular diseases, and even certain types of cancer [1,4].

Lighting plays a crucial role in the entrainment of the circadian system. Exposure to light, particularly blue-enriched short-wavelength light, during the biological night can suppress the production of melatonin, a key hormone involved in the regulation of the sleep-wake cycle [5,6]. This suppression can lead to disruptions in the circadian rhythm and associated health problems. Conversely, exposure to appropriate lighting during the day can help maintain a healthy circadian rhythm and promote various physiological and cognitive benefits [7].

In the context of nursing homes, residents often experience disruptions in their circadian rhythms due to a variety of factors, including reduced exposure to natural light, irregular sleep-wake patterns, and the use of artificial lighting that may not be optimized for circadian entrainment [8,9]. These disruptions can have significant negative impacts on the health and well-being of nursing home residents, leading to an increased risk of sleep disorders, cognitive impairment, and other chronic health conditions [9]. Recent studies have further highlighted the importance of circadian-effective lighting interventions in improving sleep quality, circadian rhythms, and depression in older adults with dementia [10-12].

Moreover, researchers have emphasized the need for tailored lighting solutions that consider the specific needs and characteristics of the nursing home population. For example, Figueiro et al [13,14] have discussed the importance of circadian lighting for older adults with dementia, as well as explored the role of light and circadian rhythms in this population.

### Personal Circadian Lighting Exposure

Assessing individual circadian lighting exposure has become increasingly vital in the health care sector, especially in long-term care facilities, where individuals’ circadian rhythms can be profoundly affected by their lighting conditions. In recent years, there has been an increasing focus on the advancement of wearable sensor technologies capable of precisely and conveniently quantifying an individual’s light exposure throughout the day. This is crucial for comprehending the effects of lighting on circadian rhythms, hormonal balance, and overall health and wellness.

The significance of precisely quantifying individual circadian lighting exposure in nursing homes is paramount. Multiple studies have underscored the significant impact of lighting on the physical and mental health of older adults, especially those with dementia. A study by Figueiro et al [15] demonstrated that customized lighting interventions in long-term care facilities resulted in notable enhancements in sleep, depression, and agitation among individuals with dementia. This highlights the necessity for precise and individualized lighting exposure data to guide the design and execution of these interventions.

Nonetheless, the existing spectrometer-based methods for quantifying individual circadian lighting exposure are frequently unfeasible for extensive application in nursing homes and other health care environments. These devices are generally cumbersome, expensive, and labor-intensive, rendering them inappropriate for prolonged monitoring and ongoing data collecting. Markvart et al [16] underscored the difficulties in calibrating and rectifying the output from wearable light exposure devices, stressing the necessity for more efficient but effective measuring technology.

### Proposed Work

In this study, we developed predictive models to assess personal circadian lighting exposure using wearable lighting sensors. These sensors measured key lighting parameters, including correlated color temperature (CCT), photopic lux, and irradiance levels at the RGB channels. The calibration of these measurements was meticulously conducted through controlled laboratory experiments under various electrical lighting conditions, supplemented by on-site data collection using professional instruments to ensure accuracy. The predictive models, built upon these calibrated lighting parameters, were designed to predict circadian melanopic lux, melanopic equivalent daylight illuminance, and circadian stimulus (CS) values. To demonstrate the practical application of these wearable sensors and the developed models, we employed them in a bright light therapy project within 2 nursing homes. Here, we analyzed variations in circadian lighting exposure across different individuals, showcasing the models’ effectiveness in capturing personal circadian light (CL) environments and their potential impact on health and well-being.

### Scientific Contributions

This work makes significant scientific contributions by introducing calibration and predictive models built upon machine learning algorithms and rigorous datasets, which effectively replace the costly and complex processes of spectrometer measurement and circadian lighting calculations. These models enable accurate prediction of circadian melanopic lux and CS values via basic lighting measures, making long-term monitoring of personal circadian lighting exposure feasible with wearable sensors.

Furthermore, by showcasing the application of these models in real-world settings, the study highlights the significant variability in CL exposure among individuals, underscoring the importance of personalized lighting assessments.

Finally, by facilitating wearable technology for CL assessment, this study paves the way for more accessible and practical approaches to understanding and optimizing light environments for individual circadian health and well-being, particularly in contexts such as nursing homes where continuous monitoring is crucial.

### Literature Review: Current Circadian Lighting Measurement Methods

Accurate measurement of CL exposure is essential for understanding the impact of lighting on the human circadian system and for developing effective lighting interventions. One of the primary instruments used for measuring CL exposure is the spectroradiometer, which is capable of providing detailed spectral information about the light environment [17,18].

Spectroradiometers are sophisticated instruments that can measure the spectral power distribution (SPD) of light, which is the relative energy of the different wavelengths of light present in a given environment. This information can be used to calculate various metrics related to CL exposure, such as the CL adjusted (CL_A_) and the CS [19,20].

In addition to spectroradiometers, other instruments such as wearable light sensors and calibrated digital cameras have also been used to measure CL exposure [17,18,21]. These methods offer the advantage of being able to capture light exposure data over an extended period, providing a more comprehensive understanding of an individual’s CL exposure patterns.

Predictive modeling approaches have also been developed to estimate CL exposure based on various environmental and personal factors. For example, models have been proposed that use information about the location, time of day, and weather conditions to predict the spectral composition and intensity of light in a given environment [7,22]. These models can be particularly useful in situations where direct measurement of light exposure is not feasible, such as in large-scale studies or in environments where spectroradiometers are not available.

In the context of nursing homes, the use of wearable sensors and predictive modeling techniques can be particularly valuable for assessing the CL exposure of residents. By combining these methods with the data obtained from spectroradiometers, researchers and health care professionals can gain a more comprehensive understanding of the lighting conditions in nursing homes and develop targeted interventions to optimize circadian entrainment and improve the health and well-being of residents [17,23].

### Wearable Sensors for Circadian Lighting Measurements

Recent years have witnessed initiatives to create more compact, cost-effective, and user-friendly wearable sensor devices for tracking personal circadian lighting exposure. These devices seek to address the shortcomings of conventional spectrometer-based solutions, offering a practical and accessible method for evaluating an individual’s light exposure throughout the day.

A primary difficulty in the development of wearable sensor systems is the compromise between measurement precision and user comfort. Figueiro et al [17] evaluated the efficacy of 3 practical field devices for measuring individual light exposures and activity levels, underscoring the necessity for more adaptable and precise wearable sensor technologies. The contemporary generation of spectrometer-based systems frequently compromises wearability because of their dimensions and measurement principles, in addition to being expensive and labor-intensive to operate.

Researchers are investigating alternate methods for measuring human circadian illumination exposure to tackle these problems. A recent work by Wang et al [24] introduced an innovative wearable sensor device that integrates a tiny light sensor with a microcontroller and wireless communication features, facilitating continuous and discreet monitoring of an individual’s light exposure. This integrated sensor system has the potential to address the shortcomings of conventional spectrometer-based solutions, facilitating broader implementation in nursing homes and other health care environments.

Kubicki et al [25] created a wearable sensor platform that integrates numerous light sensors and a machine learning algorithm to assess circadian-effective light exposure, offering a more precise and user-friendly option than current systems. This advanced sensor technology, together with predictive modeling and data analysis, enables health care providers to comprehend the correlation between lighting and the health and well-being of nursing home residents more effectively.

Emerging sensor technologies may provide exciting opportunities to assist people with dementia and their carers in the context of an aging population. Lu et al [26] provided an in-depth examination of how wearable health devices can noninvasively measure vital signs, activity, and sleep in people with vulnerabilities, such as cognitive impairment. Such technologies offer the advantage of being passive (ie, no active engagement necessary on the part of the person with dementia) and are capable of continuous data collection. On this basis, Tiersen et al [27] used user-centered design to design smart home sensing solutions for dementia households, highlighting the need for personalized monitoring solutions that address the issues both patients and caregivers face in their day-to-day life.

Furthermore, Rose et al [28] addressed the emotional and psychological consequences of the introduction of such technologies and reported family caregivers’ overall acceptability of using digital technology to record stress, but significant concerns were expressed about privacy invasion and technical complexity. Taking these concerns head-on, Berridge et al [29] created a new self-administered decision support tool to facilitate early advance planning for technology use in dementia care, allowing people in the early stages of cognitive impairment to continue to communicate their preferences about future monitoring and assistance technologies before declining to more advanced impairment. Together, these studies provide evidence that, albeit advantages of sensor technologies for monitoring and providing services to individuals with dementia, implementation should take into account user preferences, safeguarding the patient’s privacy, and integrating sensors in existing care practices.

## Methods

### Ethical Considerations

The study was approved by the institutional review board at The Pennsylvania State University (hereinafter referred to as “Penn State”) (STUDY00020216). Resident participants were assessed for their capacity to provide informed consent using the evaluation to sign consent form. Informed consent was obtained from all residents who were deemed capable. For all residents, regardless of their ability to consent, consent was also obtained from their legally authorized representatives. Resident participants did not receive compensation. However, nursing home staff who contributed information related to residents’ mood and behavioral assessments received a US $25 gift card per visit. All hard copy documents containing identifiable information were stored in locked, secure cabinets accessible only to institutional review board–approved research team members. Electronic research data were deidentified and stored on a secure research drive at Penn State, accessible only to authorized members of the research team.

### Experiments and Data Collection Procedure

To obtain comprehensive and sufficient datasets (Multimedia Appendix 1) for subsequent calibration and modeling, we conducted both laboratory experiments and field data collection in 2 assisted-living facilities. The laboratory experiments and data collection were completed at the Penn State Architectural Engineering Lighting Laboratory.

**Figure 1.**
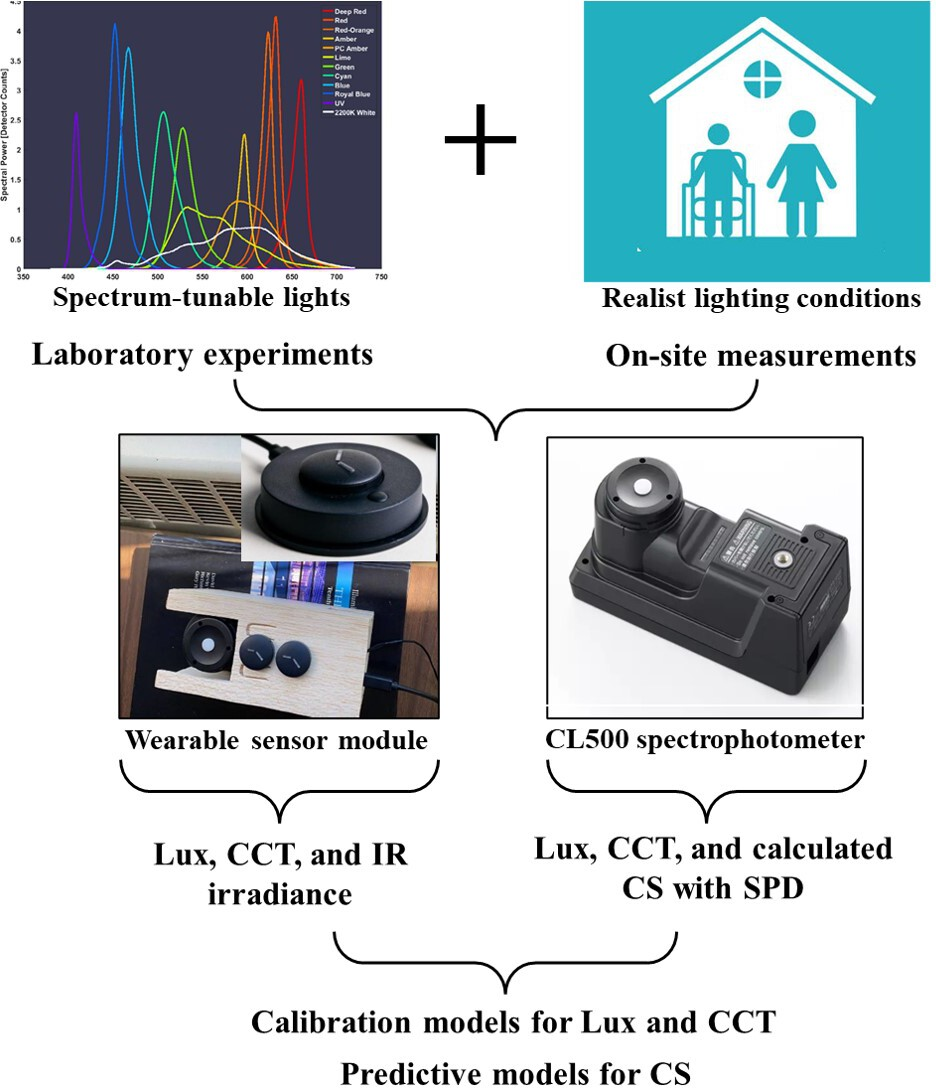
Diagram for experiments and data collection. CCT: correlated color temperature; CS: circadian stimulus; IR: infrared; SPD: spectral power distribution.

Using a professional spectrophotometer, we obtained ground truth data by placing 3 randomly selected wearable sensors and the spectrophotometer in the same position in the laboratory under various electrical lighting conditions. Moreover, to ensure the accuracy and reliability of using such models in assisted living facilities, we also conducted a similar procedure and setup in 2 selected nursing homes. In the laboratory, we simulated relatively more controllable lighting scenarios compared with the field, where we conducted several site visits on different days to capture variations related to weather, room conditions, and interior setup. The same set of instruments, wearable sensors, methods, and experimental setup was used for both laboratory and field measurements. Figure 1 shows the diagram of the major instruments used in this study. The spectrophotometer (CL500) output for photopic lux and CCT served as the reference for calibrating the wearable sensors through regression models. Additionally, the spectrophotometer’s SPD data were used to calculate CL_A_ and CS, which then acted as ground truth data for building predictive models based on the wearable sensor data. Detailed information on the instruments, experimental setup, lighting conditions, and metrics is provided in the subsequent sections. It is of importance to note that both laboratory lighting scenarios and real-world lighting scenarios included daylighting, due to its direct impact on human biology as one of the main themes for our experiment.

#### Wearable Light Sensor

In this study, we adopted the LYS Button as one of our data collection tools. The LYS Button is incorporated with the LYS app, enabling users to collect data on individual daily CL exposure. The LYS wearable button is a compact and lightweight device and is ideal for individuals such as our human participants and assisted-living residents impacted by mild to moderate dementia, who tend to not respond well to large objects hanging off their clothing. The wearable sensor is an ideal tool when optimizing lighting environments. The LYS Button can track various parameters including photopic illuminance, color temperature, irradiance at R (465 nm), G (525 nm), B (615 nm), and infrared (IR) (1100 nm) channels, and motion. The key features of this wearable sensor are shown in Table 1. One of the drawbacks of the LYS Button is that it does not directly output circadian lighting levels, such as melanopic lux and CS. The availability of light data through the R, G, and B channels using the LYS device, which can be converted to melanopic data, was of importance and value, hence why it was selected for this study.

**Table 1.** LYS Button: key features.

Feature	Specification
Illuminance measurement	0.1-100,000 lux
Color temperature measurement	1800-10,000 K
Continuous monitoring and data logging	Enabled
Connectivity	Bluetooth to mobile app
Battery life	Up to 7 days per charge
Design	Compact, discreet, and wearable
Companion mobile app	Data visualization and analysis

#### Spectrophotometer

CL500A Illuminance Spectrophotometer (Konica Minolta) was used In this study, as the tool to obtain the ground truth data for developing calibration and predictive models (Multimedia Appendix 2) from the wearable sensor data. It can measure illuminance, CCT, color-rendering index, and many other color or light parameters. Also, the instrument uses a silicon photodiode array and a diffraction grating to provide full SPD data, which can be then computed to obtain CL_A_ and CS. The other key features of CL500A are listed in Table 2.

**Table 2.** CL500A Spectroradiometer: key features.

Feature	Specification
Spectral measurement range	360-780 nm, 1-nm resolution
Illuminance measurement range	0.01-100,000 lx
Calculated parameters	Chromaticity coordinates, CCT^a^, and CRI^b^
Display	Large, easy-to-read color liquid crystal display
Data transfer	USB interface to a computer
Design	Compact and lightweight, portable
Calibration	Traceable to national standards

a CCT: correlated color temperature.

b CRI: color-rendering index.

### Laboratory Data Collection

#### Overview

We conducted our experiment at the Penn State University Architectural Engineering Lighting Laboratory, adhering to specified standards to ensure the precision and dependability of our results. Our methods conform to relevant international standards, including ISO 17025 for laboratory quality management. The laboratory is equipped with NIST-traceable luminaires, which allowed us to create a variety of electrical lighting conditions. Photopic lux levels ranged from 10 to 10,000, and CCT values spanned a broad range between 1500 to 10,500 K. The CCT distribution was achieved using 4 primary indoor light sources with CCT of 1500 K, 3000 K, 4200 K, and 6500 K. These sources were used individually or in combination, and—along with natural daylight entering through windows—they enabled coverage of the broad CCT range. Illuminance levels were similarly generated using these 4 dimmable light sources, allowing fine control over light intensity. Higher data density was intentionally designed and collected around 2 key illuminance ranges: one representing typical indoor artificial lighting and another corresponding to daylight-accessible conditions. As a result, we generated 100 different lighting scenarios by combining various lighting intensities and CCT conditions in the lighting laboratory. For each scenario, we waited approximately 10 minutes to ensure stability before logging 5 data points. The LYS Button wearable sensors automatically documented and uploaded the measured data to a central cloud, while the CL500A’s data were transmitted directly to a computer via cable connection.

#### National Institute of Standards and Technology

NIST-traceable luminaires, which allowed us to create a variety of electrical lighting conditions. Photopic lux levels ranged from 10 to 10,000, and CCT values spanned a broad range between 1500 to 10,500 K. The CCT distribution was achieved using 4 primary indoor light sources with CCT of 1500 K, 3000 K, 4200 K, and 6500 K. These sources were used individually or in combination, and—along with natural daylight entering through windows—they enabled coverage of the broad CCT range. Illuminance levels were similarly generated using these 4 dimmable light sources, allowing fine control over light intensity. Higher data density was intentionally designed and collected around 2 key illuminance ranges: one representing typical indoor artificial lighting and another corresponding to daylight-accessible conditions. As a result, we generated 100 different lighting scenarios by combining various lighting intensities and CCT conditions in the lighting laboratory. For each scenario, we waited approximately 10 minutes to ensure stability before logging 5 data points. The LYS Button wearable sensors automatically documented and uploaded the measured data to a central cloud, while the CL500A’s data were transmitted directly to a computer via cable connection.

**Figure 2.**
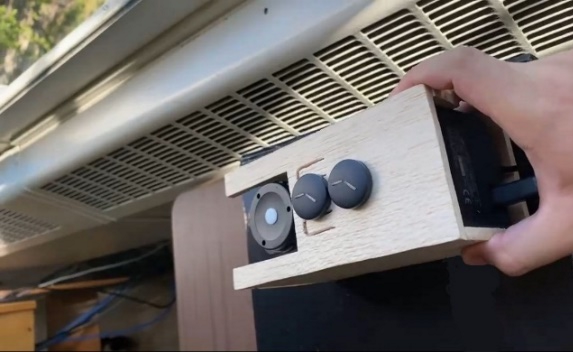
Data collection at Penn State University lighting laboratory.

We affixed 3 randomly selected wearable sensors to the CL500A spectrophotometer at the same position within the laboratory, enabling the study team to obtain concurrent readings from both instruments. This method facilitated a direct comparison of the measurements and the detection of any inconsistencies between the 2 sensors (Figure 2). The CL500A’s output for photopic lux and CCT served as reference points to calibrate the wearable sensors through regression models. Additionally, the CL500A’s SPD data were used to calculate CS and melanopic lux, which then acted as ground truth data for building predictive models based on the wearable sensor data.

### On-Site Data Collection

**Figure 3.**
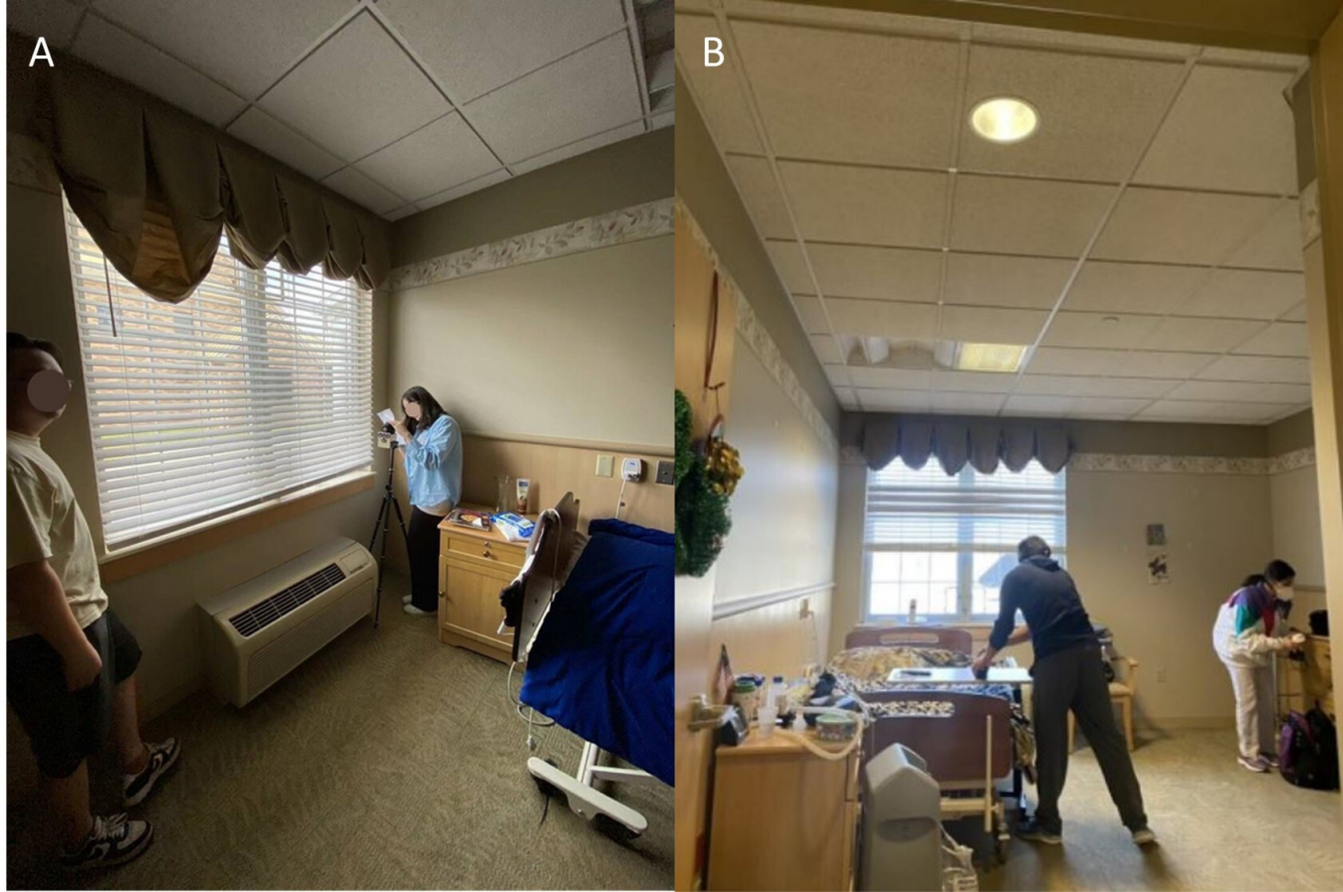
On-site data collection in the window area (A) and the living area (B) by authors at the assisted-living facility.

The on-site data collection with the CL500A and LYS Buttons was executed in different residential rooms of 2 assisted-living facilities located in Pennsylvania. We intentionally visited the nursing homes under varying conditions, such as different weather, times of day, weekdays and weekends, and room types (resident rooms, hallway, dining hall, and activity rooms). Furthermore, in each room, the measurements were conducted at various distances from the windows, spanning from 0 to 9 feet, to evaluate the devices’ reactions to varying amounts of daylight. The electrical lights were also modulated to form different daylight-electrical light combination scenarios. The window blinds were positioned at 4 distinct slat angles, 135°, 45°, and 90°, as well as the fully retracted position, to evaluate the effect of window shade on illumination conditions. The measurement stations were arranged at multiple viewing angles, ranging from 0°, directly facing the window, to 270° to assess their effectiveness across diverse orientations. The primary light sources in the 2 nursing homes consisted of 3 different CCT types: 1800 K, 2700 K, and 4000 K. Through controlled switching of these sources and adjustment of blinds to modulate daylight access, a broad range of illuminance and CCT variations was achieved. Similar to the laboratory settings, illuminance levels were primarily concentrated in lower ranges typical of dim indoor environments without daylight and in moderate ranges when daylight was present. CCT values were predominantly in the lower spectrum due to the warm indoor lighting but extended to higher levels when daylight contributed to the scene. All these steps and setups aimed to capture sufficient variations in lighting conditions from the window zone (Figure 3A) and the main bed area (Figure 3B) for subsequent calibration and prediction as displayed.

### Circadian Metrics Used for Data Processing

CS, closely associated with CL_A_, is a metric that directly quantifies the extent of circadian system activation resulting from specific light exposure [20]. Because neither spectrophotometers (CL500A) nor wearable sensors (LYS Button) can output CS directly, we had to extract the raw spectral data from CL500A and then input them into the CS calculator developed by Rensselaer Polytechnic Institute [19,30-32] as seen in equation 1. CS [33], shown in equation 2, is derived from the CL_A_ value.

. (1) $$\begin{array}{c} CLA=1548\left\{\int Mc_{\lambda }E_{\lambda }\;d\lambda +a_{b-y}\left(\int \frac{S_{\lambda }}{{mp}_{\lambda }}E_{\lambda }\;d\lambda -k\int \frac{V_{\lambda }}{{mp}_{\lambda }}E_{\lambda }\;d\lambda \right)-\;a_{rod}\left(1-e^{\frac{-\int v_{\lambda }E_{\lambda }\;d\lambda }{RodSat}}\right)\right\},\;b-y§gt;0\\ 1548\int M_{c\lambda }E_{\lambda }\;d\lambda ,\;b-y\leq 0\\ where,\\ b-y=\int \frac{S_{\lambda }}{mp_{\lambda }}E_{\lambda }\;d\lambda -0.2616\int \frac{V_{\lambda }}{mp_{\lambda }}E_{\lambda }\;d\lambda \end{array}$$

(2) $$CS=0.7*\left[1-\frac{1}{1+{\left(\frac{{CL}_{A}}{355.7}\right)}^{1.1026}}\right]$$

Equation values and parameters are shown in Textbox 1.

Textbox 1. **Equation values and parameters.**

CL_A_: circadian light adjusted.

*E*_λ_: light source spectral irradiance.

*Mc*_λ_: melanopsin sensitivity (corrected for crystalline lens transmittance) [34].

*k*=0.2616

*S*_λ_: S-cone fundamental.

*a*_*b*–*y*_=0.7

*mp*_λ_: macular pigment transmittance.

*a*_rod_ = 3.3

*V*_λ_: photopic luminous efficiency function [35].

RodSat = 6.5 Wm^−2^

*V*_λ′_: scotopic luminous efficiency function [35].

### Calibration and Predictive Modeling Methods

#### Calibration Modeling of Photopic Lux and CCT

The calibration process was implemented to ensure the measurement accuracy for the parameters of photopic lux and CCT from LYS. Simple regression calibration was applied first using the data obtained from the reference devices. The original data can be found in Multimedia Appendix 2. The candidate parameters from the reference devices included red, green, and blue (RGB) value, IR light, illuminance, and color temperature.  

The photopic lux was calibrated based on the illuminance measurements from CL500A and the measurements from LYS about illuminance, RGB, IR, and CCT. The measurement accuracy of LYS was calibrated for its performance under different lighting conditions. Similarly, CCT from LYS was also calibrated based on the CCT measurements from CL500A. The data screening process was first conducted to check the negative values that were invalid and to enhance the data quality. The feature selection of these candidate parameters for the simple regression model was based on their significant test results. The models were developed and evaluated using a train-test split for the assessment of modeling metrics. In the train-test split method, 70% of the total data were used as a training set to train the model, and 30% were used as test data to value the model’s performance. Evaluation metrics including *R*², mean squared error (MSE), and mean absolute error (MAE) were used to indicate the goodness of fit of the model. Equations 3-5 show the mathematical formula used for each of the implemented evaluation metrics.


(3) $$MAE=\frac{1}{N}\sum_{i=1}^{N}\left|y_{i}-\hat{y}\right|$$

(4) $$MSE=\frac{1}{N}\sum_{i=1}^{N}{\left(y_{i}-\hat{y}\right)}^{2}$$

(5) $$R^{2}=1-\frac{\sum (y_{i}-\hat{y}{)}^{2}}{\sum (y_{i}-\bar{y}{)}^{2}}$$

where $\hat{y}$ is the predicted value of y, and $\bar{y}$ is the mean.

#### Predictive Modeling of CS

The prediction models were developed based on the available parameters’ information for the CS from CL500A. The original data can be found in Multimedia Appendix 2. The calculation of CS has been studied in diverse ways for quick and efficient computation. The LRC Circadian Light Combined Calculator was applied in the study in section “Circadian Metrics Used for Data Processing.” The calculator was programmed to enable the output of CS by the input of SPD for a lighting source. The spectral lighting with the wavelength from 360 to 780 nm could be obtained from the CL500A and thus the values of CS were obtained using the CS calculator. The spectral lighting data with a 1-nm interval were first integrated into a 5-nm increment for an efficient computation in the calculator.

After the calculation of the CS, a CL prediction model was developed based on the measured values from LYS including RGB value, IR light, illuminance, and color temperature. The simple regression model was applied first based on the train-test split to check the prediction accuracy using evaluation metrics of R2, MAE, and MSE. To better evaluate the reliability of a prediction model, especially for the data collected under different lighting environments, the 5-fold cross-validation shown in equation 6 was further used to identify the optimal simple regression model.


(6) $${CV}_{\left(k\right)}=\frac{1}{k}\sum_{i=1}^{n}{error}_{i}$$

where the error can refer to evaluation metrics such as MAE, MSE, and *R*².

To achieve high and satisfactory prediction accuracy, a random forest model was used for predictive analysis if the simple regression model did not meet the desired performance criteria. A random forest model enabled a multitude of decision trees during training and output the mean prediction for regression of the individual trees. This bagging technique, where different subsets of the dataset are used to train each decision tree, made the overall model more robust and less prone to overfitting. Compared with a linear regression, it could provide a comparison of the feature importance which can contribute to the understanding of the influence of the input variables on the output.

## Results

### Calibration Model for Photopic Lux

**Figure 4.**
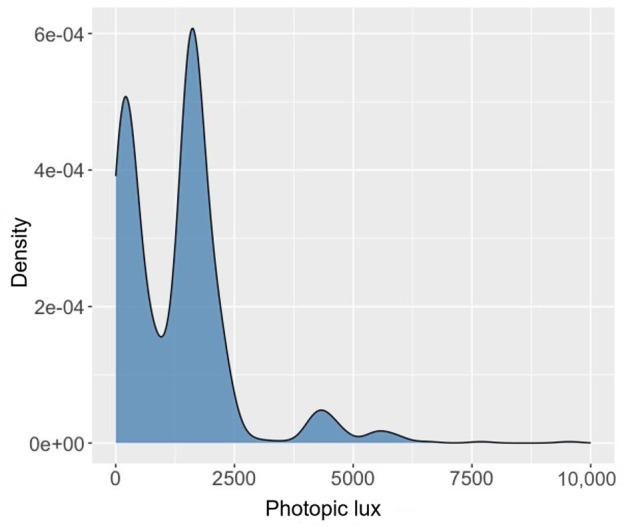
Photopic lux distribution during data measurement.

Figure 4 shows the distribution of photopic lux measurements collected from our experiments at both the laboratory and on-site. The data covered a range from ultra-low to high lighting levels, corresponding to 2 main locations. The first peak (at lower lux values) likely represented interior spaces illuminated by artificial lighting, while the second peak (at higher lux values) corresponded to areas near windows with natural daylight exposure.

For the photopic lux calibration, a simple regression model with acceptable accuracy was obtained. The input parameters, including the LYS Button’s output, which include CCT (Kelvin), IR channel irradiance, and illuminance (lux), were selected during the modeling analysis. The calibration function is shown in equation 7. This calibration function adjusts the original measurements from the LYS Button (CCT’, IR, and lux’) to more accurately reflect true photopic lux (Y). The coefficients (0.0596, 1.6064, and 0^.^8432) represent the contribution of each parameter to the final calibrated value.

 (7) $$Y\left(Photopiclux\right)=0.0596*{CCT}^{\prime }+1.6064*IR+0.8432*lux$$

**Figure 5.**
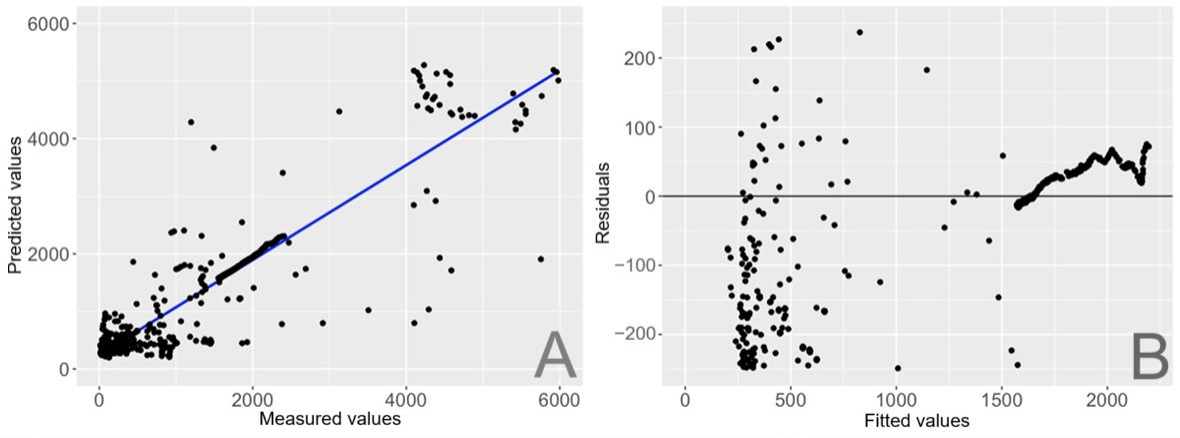
Calibration results for photopic lux: (A) Predicted versus measured values. (B) Residuals versus fitted values.

Where CCT’, IR, and lux’ refer to the original output from the LYS Button—CCT (Kelvin), irradiance of near-IR wavebands at the peak of 1100 nm (W/m²), and illuminance level (lux), respectively—the comma differentiates the final calibrated parameters, CCT, and lux. This may be due to small changes in solar light or daylight, which can significantly boost the lux measurement, as near-IR sources are primarily from daylight. In contrast, the original lux’ measurement has a smaller coefficient, suggesting that it was initially less accurate and thus required a substantial adjustment. The CCT’ shows a minor but necessary adjustment. Together, these coefficients correct the original sensor outputs to better match the true photopic lux values. The overall adjusted *R*² and MAE for this calibration model are 0.858 and 355, respectively. The goodness-of-fit plot is shown in Figure 5 (A), indicating that the predicted values generally correspond to the measured values. Plot (B) in Figure 5 shows the residuals against the fitted values, in which the residuals are widely dispersed especially at lower fitted values. However, at the higher fitted value range, there is a clear pattern, possibly suggesting model misspecification on the presence of nonlinear relationships. However, for general indoor lighting conditions, the calibration model shows a general trend of aligning with the measured values.

### Calibration Model for CCT

**Figure 6.**
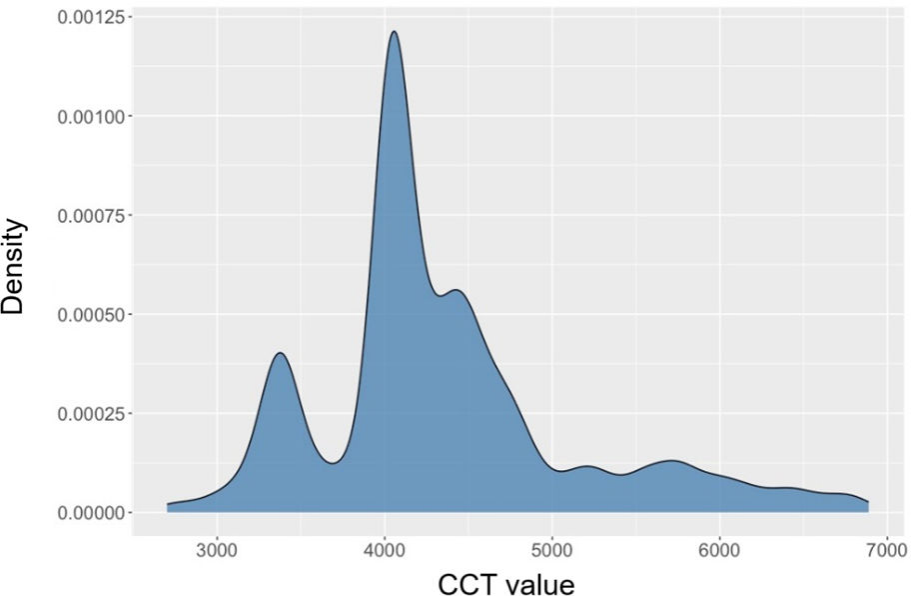
Correlated color temperature distribution during data measurement. CCT: correlated color temperature.

Figure 6 illustrates the distribution of CCT values recorded during the data collection procedure. Similar to the distribution of photopic lux, it represents 2 key environments. The initial peak (around 3000‐4000 K) likely corresponds to artificial interior lighting with a warmer color temperature, while the subsequent distribution (around 4000‐5000 K) may represent areas near windows with daylight, featuring a cooler color temperature.

Similar to the calibration model of photopic lux, for the CCT calibration, a simple regression model with satisfactory accuracy was also obtained. The input parameters, including the LYS Button’s output—CCT (Kelvin), IR channel irradiance, and illuminance (Lux)—were selected again during the CCT calibration model development. The calibration equation is shown in equation 8:


(8) $$Y(CCT)=1.048\times CCT^{\prime }-0.5427\times IR+0.1761\times lux^{\prime }$$

**Figure 7.**
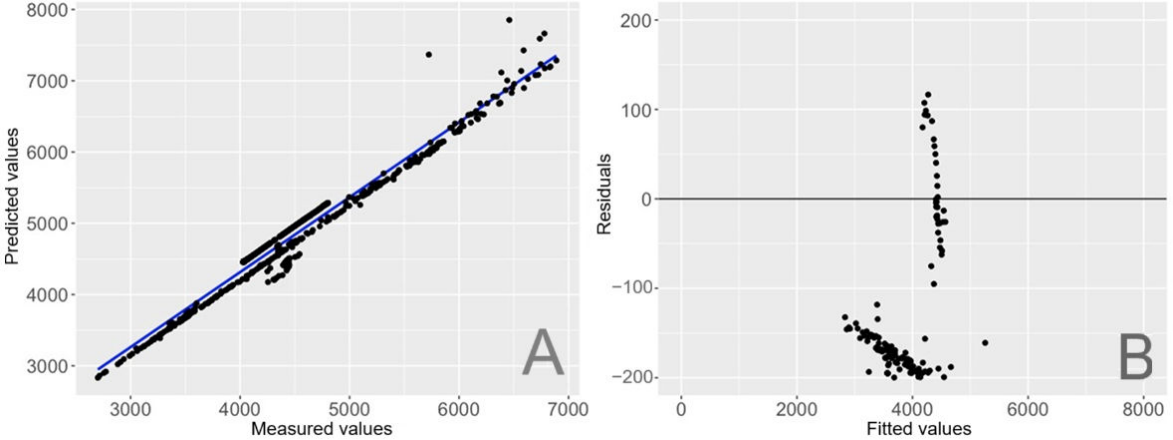
Calibration for correlated color temperature. (A) Predicted versus measured values. (B) Residuals versus fitted values.

In the calibration model, the Kelvin component, with a coefficient of 1.025, shows a strong correlation with the true CCT, indicating that the original CCT values are quite close but require a slight adjustment. The IR component has a relatively strong negative influence on the CCT, highlighting the significant impact of solar IR on the calibration process, likely due to its effect on perceived color temperature, particularly from daylight sources. Together, these coefficients adjust the original sensor outputs to better match the true CCT values. The overall adjusted *R*² and MAE for this calibration model are 0.982 and 342, respectively. The goodness-of-fit plot is shown in Figure 7A, and the model bias and variance patterns in Figure 7B.

### Evaluation of Predictive Models for CL_A_

**Figure 8.**
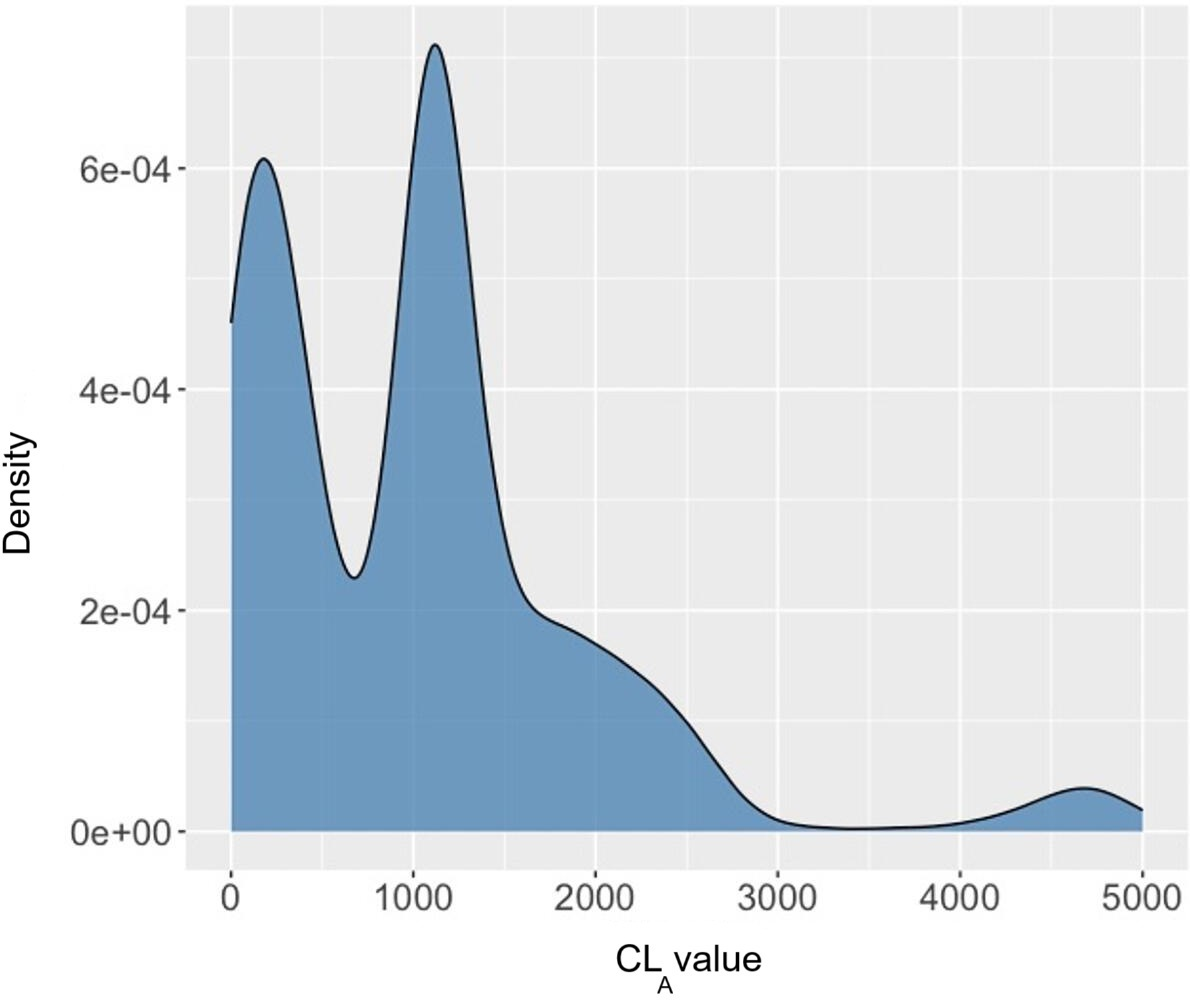
Circadian light adjusted (CLA) distribution during data measurement.

Figure 8 represents the distribution of CL_A_ values collected, which also reflects 2 major lighting conditions. The first peak (at lower CL_A_ values) likely corresponds to areas with artificial lighting, providing minimal circadian stimulation. The second peak (at higher CL_A_ values) likely represents zones near windows with natural daylight, which contribute more significantly to CL_A_ exposure. The remaining distribution tail illustrates sporadic occurrences of very high CL_A_ values, likely in brightly lit areas with substantial daylight, which is also aligned with the photopic lux data distribution.

Based on the modeling procedure illustrated in section “Laboratory Data Collection,” we obtained both simple regression models and machine learning–based models. As shown in Table 3, the machine learning–based model shows much more accurate performance relative to the linear regression model, with an adjusted *R*^2^ of 0.86.

**Figure 9.**
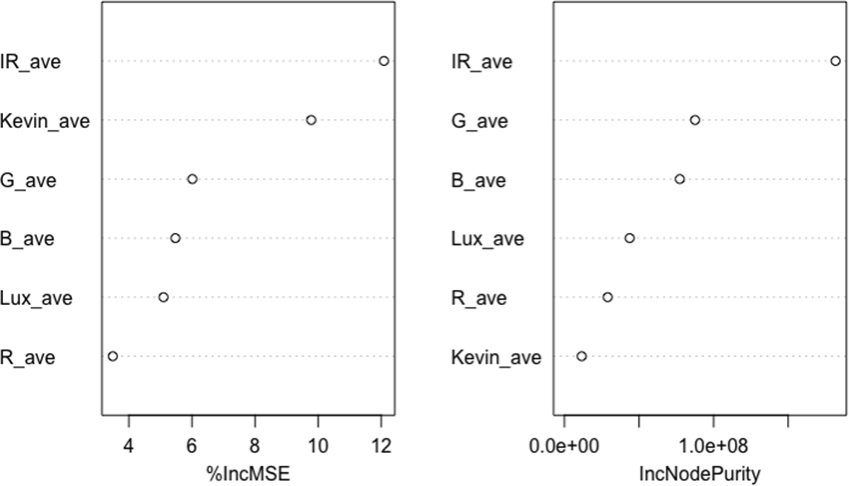
Variable importance.

Regarding the machine learning models, we also conducted the importance feature ranking analysis. Figure 9 shows the variable importance ranking results using 2 metrics %IncMSE (percentage increase in MSE) and IncNodePurity (increase in Node Purity). %IncMSE measures the increase in the model’s prediction error when a particular variable is randomly permuted while all others are kept constant. A higher value indicates greater importance, while IncNodePurity is based on the total decrease in node impurities contributed by a variable across all the trees in the forest. As shown in this context, the IR value has the highest in both metrics consistently, which is similar to the calibration model results in CCT and photopic Lux calibration—the potential daylight effects on indoor IR levels drive the variations of circadian lighting level changes. The sensor’s original CCT value had a strong direct impact on the model’s predictive accuracy, while it might not be crucial for creating pure or homogenous nodes, meaning it did not contribute much to the structure of the decision trees.

**Table 3.** Model comparison.

Modeling techniques	Performance	MAE^a^	RMSE^b^
Random forest	0.8567 (cross-validation *R*^2^)	180.53	645
Linear regression	0.645 (adjusted *R*^2^)	491.5	1050

a MAE: mean absolute error.

b RMSE: root-mean-square error.

### Applications in Circadian Lighting Health Research in Nursing Homes

To understand the functionality and usage of the wearable sensors with calibrated and predicted models, we applied the sensors to 29 older adults with dementia in 2 assisted-living facilities as part of a National Institutes of Health–supported project. A post hoc power analysis using GPower 3.1 [36] indicated that with a total sample size of 29 and α=.05, the study had 87 % power [37] to detect a medium effect size (Cohen *d*=0.53), as reported in the previous studies [14,15]. During the personalized data collection from residents, the LYS device was pendant-worn or clipped on the upper body area (eg, upper chest, shirt collar, and shoulder) to more closely approximate eye light exposure. Staff and nurses have been guided to make sure that the attachment of devices is in secure positions as well as constant monitoring of the residents to ensure that the device has not been accidentally removed from the human participant. Researchers also visited sites twice a week to ensure the sensor attachment. Additionally, the LYS device supports real-time remote monitoring. We implement automated algorithms on the cloud to detect potential nonwear or malfunction events (eg, no change in light and motion data readings for more than 2 hours during the daytime and 4 hours during the nighttime). When such events were identified, alerts would be sent to the research team who would follow up with on-site verification and resolution as needed.

The sensor data for each individual was automatically uploaded to a central computer (Mac Mini) located in the dining hall of the nursing homes to ensure Bluetooth connectivity to all participant residents and then retrieved through a remote connection. By using the calibrated and predicted sensor measurement methods developed in this work, we were able to analyze each resident’s circadian lighting exposure, enabling more rigorous and effective intervention analysis.

We then selected specific residents with the widest range of variabilities and differences to demonstrate the applicability of our developed methods within the following sections. It is important to note that any analysis shown in this section is of exploratory value; the relatively small focus of the study in this section was to illustrate the measurement methodology for potential use in future studies on circadian lighting health.

### Intervariability of Residents’ Lighting Exposures

Understanding the intervariability of residents’ lighting exposures is crucial for accurately analyzing the effects of interventions, as it accounts for the differences in physical environments and personal factors among individuals. These 2 residents were particularly selected due to their differences in the dementia stages, the room’s window orientation direction, and daily routine and schedules. To do this, we particularly extracted 2 residents’ hourly sensor data and then calculated weekly averages of photopic lux, CCT, and CS. Resident 1’s room faced south, and resident 2’s faced east. Resident 1 had mild dementia, and resident 2 had moderate dementia. It was also observed that resident 1 benefited from the facility’s activities such as spending several hours during the day in the sun-lit dining area and having scheduled outdoor visits more frequently than resident 2. Table 4 denotes key differences between the 2 residents selected for detailed analysis in this study, providing context for the observed variations in CS, lux, and CCT measurements in the subsequent sections.

**Figure 10.**
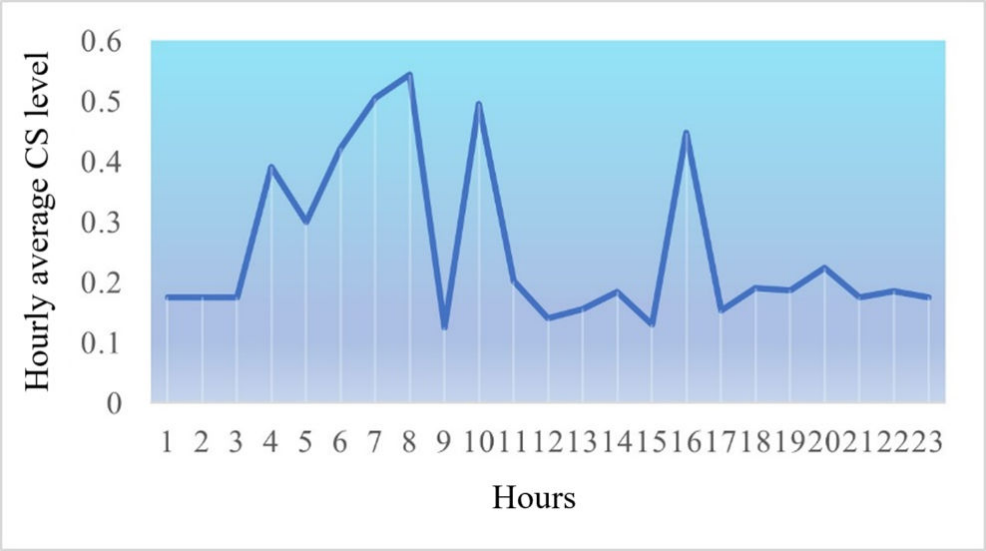
Hourly circadian stimulus intravariability. CS: circadian stimulus.

Figures10^12^ are the detailed comparisons in terms of their variations in CS, Lux, and CCT based on our calibrated and predicted measurement models, using a total of 268 data points collected across 5 project weeks and processed using the machine learning and regression models in section “Calibration and Predictive Modeling Methods” in R Studio [Posit] and visualized using Python features (the main coding information used in this work can be found in Multimedia Appendix 1).

**Table 4.** Comparative characteristics of selected study residents.

Characteristic	Resident 1	Resident 2
Dementia stage	Mild	Moderate
Room orientation	South-facing	East-facing
Activity level	High; regular participation	Limited; infrequent participation
Outdoor access	Frequent scheduled visits	Minimal exposure
Daily routine	Structured; time in sun-lit areas	Less structured; more time in room
Sleep pattern	Generally stable	Erratic; disrupted
Light exposure	Consistent throughout day	Morning-dominant
Daytime behavior	Alert; minimal sundowning	Variable alertness; evening agitation

**Figure 11.**
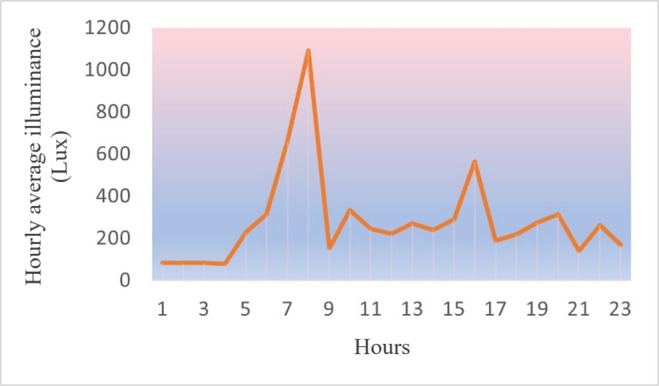
Hourly illuminance intravariability.

**Figure 12.**
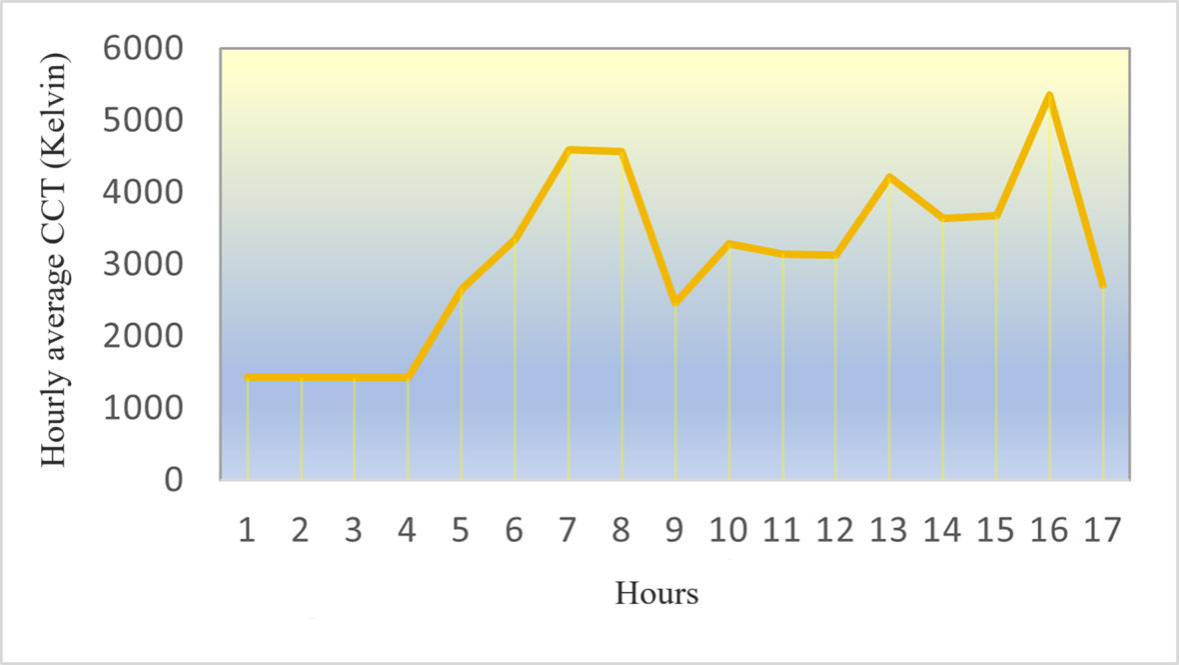
Hourly daytime correlated color temperature variations. CCT: correlated color temperature.

#### CS Intervariability

Multimedia Appendix 3 visualizes the weekly daytime and nighttime CS value variations of the selected 2 residents across the project. When comparing nighttime CS levels between resident 1 (mean 0.196, variance 0.00223) and resident 2 (mean 0.228, variance 0.00167), the paired *t* test analysis revealed no statistically significant difference between the 2 residents (2-tailed *t*^_4_^=−1.01; *P*=.368). Although resident 2 showed a slightly higher mean nighttime CS value, this difference was not substantial enough to reach statistical significance at the α level of .05. Analysis of daytime CS levels revealed that resident 1 (mean 0.414, variance 0.00418) demonstrated marginally higher values than resident 2 (mean 0.326, variance 0.00113). This difference approached statistical significance in the paired *t* test (2-tailed *t*^_4_^=2.43; *P*=.072). While not meeting the conventional *P*<.05 threshold for statistical significance, this trend suggested that resident 1 potentially experienced greater circadian-effective light exposure during daytime hours than resident 2.

Both residents have higher CS levels during the daytime compared with nighttime throughout the period. Comparatively, across those weeks, resident 1 with moderate dementia generally had higher daytime CS levels and lower nighttime CS levels than resident 2 in most weeks, with the highest average daytime CS of 0.5 for resident 1 during week 5 and 0.38 for resident 2 during week 2, and the highest average nighttime CS of 0.29 for resident 2 during week 5 and 0.24 for resident 1 during week 4. The differences in both daytime and nighttime CS levels between the 2 residents can be attributed to several factors, in particular their different dementia stages, different daytime activity participation levels, and bedroom orientations as pointed out in section “Intervariability of Residents’ Lighting Exposures.”

Resident 2 with moderate dementia (reported erratic nighttime behaviors and activities) living in an east-facing room and lower daytime CS levels had less participation in the facility’s activities and as a result had reduced daylight exposure. As expected, resident 2 displayed higher nighttime CS due to disrupted sleep. Their east-facing orientation provided less consistent and intense daylight than the south-facing room of resident 1, impacting the overall daytime and nighttime CS-level variations between the two.

#### Lux Intervariability

With regard to weekly daytime and nighttime illuminance level comparisons, there were notable differences between the 2 residents. For nighttime illumination levels, statistical analysis revealed a significant difference between resident 1 (mean 141.58 lux, variance 2558.22) and resident 2 (mean 233.58 lux, variance 4042.12), with the paired *t* test showing statistical significance (2-tailed *t*^_4_^=−2.78; *P*=.050). This finding indicates that resident 2 experienced significantly higher illumination levels during nighttime hours than resident 1, which aligns with observations of resident 2’s reported sleep disturbances and increased nighttime activity. For daytime illumination levels, data analysis comparing resident 1 (mean 422.24 lux, variance 4438.70) and resident 2 (mean 376.36 lux, variance 493.39) showed no statistically significant difference (2-tailed *t*^_4_^=1.32; *P*=.258). While resident 1 experienced somewhat higher average daytime illumination levels, the considerable variance in resident 1’s measurements and the relatively small sample size likely contributed to the nonsignificant finding. This suggests that both residents received comparable amounts of light exposure during daytime hours despite their different room orientations and activity patterns.

As seen in Multimedia Appendix 3, resident 1, who had a milder dementia stage than resident 2 and resided in a south-facing bedroom, was exposed to a sufficient amount of daytime light exposure. This abundance of natural light not only supported resident 1’s circadian rhythms, as discussed in section “Intervariability of Residents’ Lighting Exposures,” but also had the potential to positively impact their mood, alertness, and overall well-being according to the observations. In contrast, resident 2, who had a moderate dementia stage and an east-facing bedroom, experienced slightly lower daytime lux levels than resident 1. However, it is important to note that resident 2’s room would still receive natural light exposure, particularly during the early morning hours when the direct sunrise exposure provides a high level of illumination, as both residents ultimately had sufficient average amounts of lux close to 400 per recommendations and standards.

It is worth mentioning that the differences in daytime lux levels between the 2 residents can also be attributed to their varying levels of participation in daily activities and programs. Resident 1, with a lower dementia stage, was likely more engaged in general activities scheduled by the facility, which made a further contribution to their increased exposure to daytime light, due to the activity area being sun-lit from morning to afternoon. In contrast, resident 2, with a moderate dementia stage, may have had reduced participation in activities and programs, leading to slightly lower daytime lux levels. Resident 2 also had significantly higher nighttime illuminance exposure due to erratic sleep patterns.

#### CCT Intervariability: Accuracy Challenges of CCT Measurements in Dim Conditions

A primary obstacle in quantifying CCT in low-light conditions is the intrinsic difficulty in precisely measuring the SPD of the light source. Rea and Freyssinier [38] emphasize that under low-light situations, the spectral sensitivity of the human eye alters, resulting in possible disparities between the measured CCT and the perceived CCT. This may lead to mistakes when using conventional colorimetric systems, which are calibrated for elevated light levels. Chen et al [39] examined the efficacy of different CCT measurement methods in low-light conditions, showcasing that these challenges are especially evident in low-light conditions, where the signal-to-noise ratio of the measurement may be considerably diminished. Li et al [40] assert that conventional CCT calculation methods may be inadequate for these light sources, as they can display intricate SPDs that may result in erroneous CCT estimations. Owing to these constraints, we exclusively examined the daytime CCT to align more closely with reality.

Analysis of nighttime CCT levels revealed a notable difference between resident 1 (mean 2098.10 K, variance 515642.97) and resident 2 (mean 3733.34 K, variance 1760014.09), with results approaching statistical significance (2-tailed *t*^_4_^=−2.26; *P*=.086). This trend suggests that resident 2 was exposed to lighting with a substantially cooler color temperature during nighttime hours compared with resident 1, which may help explain some of the observed differences in nighttime behavior and sleep quality, as cooler (bluer) light is known to potentially disrupt circadian rhythms and melatonin production during evening hours. Examination of daytime CCT values revealed no statistically significant difference between resident 1 (mean 4219.44 K, variance 226319.65) and resident 2 (mean 3880.88 K, variance 520702.89) according to the paired *t* test analysis (2-tailed *t*^_4_^=0.81; *P*=.465). While resident 1 experienced slightly higher color temperature exposure during daytime hours, suggesting some exposure to cooler, more bluish light that typically enhances alertness, the substantial variance in measurements and small sample size resulted in a nonsignificant finding. This indicates that both residents generally experienced comparable light spectral qualities during daytime, despite differences in room orientation and activity patterns.

With regard to the CCT variations seen in Multimedia Appendix 3, resident 1 tended to experience a slightly higher daytime CCT than resident 2. This can be primarily attributed to the consistent natural light exposures that resident 1 receives for a longer duration throughout the day, due to the south-facing orientation of their room. The increased exposure to this cooler, bluer-toned light can have beneficial effects on the resident’s circadian rhythms, cognitive function, and overall well-being.

In contrast, resident 2’s east-facing room receives less direct sunlight exposure throughout the day, leading to a relatively lower daytime weekly average CCT. While resident 2’s daytime CCT levels are lower than those of resident 1, it is important to note that the early morning sunlight coming from the east, even with a lower CCT, can still help entrain the resident’s sleep-wake cycle and promote better synchronization with the natural light-dark cycle. It is seen that resident 1’s higher average levels of CCT are also partly due to their participation in outdoor activities, while resident 2 did not participate as frequently.

In sum, the analysis of lighting exposure intervariability among residents, using the regression and machine learning models developed in this study, may provide help to identify moderating factors that may influence the effectiveness of lighting interventions across different residents.

### Intravariability of Residents’ Lighting Exposures

Examining the intravariability of each resident’s light exposure provides insights into the daily fluctuations and consistency of their lighting environments. Especially for circadian lighting intervention studies, this understanding allows researchers to understand individual characteristics and adjust lighting interventions to ensure that target conditions are maintained. From a long-term application perspective, this can also enable lighting system adjustments and personalization to maintain optimal circadian lighting exposure. As such, we particularly select 1 resident who exhibits a mild stage of dementia, with intermittent reports of midnight roaming behaviors.

The hourly data shown in Figures 10-12 were obtained from this resident on 1 weekend day in October with partly cloudy sky conditions. Their room was oriented toward the east, which allows for direct sunshine exposure from sunrise until approximately 9 AM. During the afternoon hours, this resident tended to spend more time in their bedroom, where they received minimal sunshine exposure. In particular, this specific room orientation and the resident’s daily routine help explain the observed surge in CS, lux, and CCT measurements during the morning hours.

This resident’s CS levels can be explained by their room orientation (east-facing window) as well as their daily schedule on the specific day of data collection. During the lunch and early dinner hours (before 11 AM and around 4 PM), the resident was seated in the dining room, which has large windows and receives abundant natural light throughout the day (Figure 10). Throughout the night, they had low CCT levels, which points to their uninterrupted sleep that evening.

As the sun rises, it shines directly through this resident’s east-facing room, contributing to a high level of illumination. Later in the day, around 4 PM, the resident was exposed to the direct, lower-angle rays of the setting sun on the western side of the building (Figure 11). During the evening, illuminance remained at a low level and it was in accordance with the observation of their normal sleep.

The daytime CCT for this resident seems to plummet between 4 PM and 5 PM. This occurrence was likely due to the resident stepping outside briefly scheduled by their daily routine and then going back to the interior where there were much lower CCT conditions (Figure 12). Nighttime CCT data were not processed due to the constraints mentioned in section “CCT Intervariability.”

The detailed analysis of this resident’s lighting exposure and daily activities highlights the significant role of factors such as room orientation, daily routines, and engagement in facility programs in shaping their overall circadian rhythms and well-being. The data collected showcased the potential benefits of optimizing lighting and daily schedules for residents with mild dementia to promote better engagement and awareness, and the data processing with the models we developed in this study provided insights into the relationship between their circadian lighting exposure and engagement in daily activities. These results can help health care providers, nurses, and families of older adults with dementia to ensure that they are making the necessary lighting and routine adjustments for these residents.

### Summary of Application Findings

The application here demonstrated the effectiveness of wearable sensors, combined with calibration and predictive models, in assessing individual circadian lighting exposure. By capturing personal light exposure data, these models enabled a detailed analysis of how factors such as room orientation, daily routines, and activity engagement influence circadian rhythms. This approach highlights the significant variability in lighting experiences among individuals, which traditional group-level assessments may overlook.

The small focus of the study was to validate the measurement methodology for use in future studies of health impacts. We recognize that additional validated research will be needed to perform robust effect analyses in larger and more diverse populations and to determine the broader impact on health. Such efforts can greatly enhance circadian lighting research, enabling more targeted interventions and improving data-driven decision-making in health care environments.

## Discussion

### Principal Findings

Despite its contributions, this study encountered several challenges related to sensor wearability, durability, and data collection. In the application projects, some dementia residents found the sensors uncomfortable, reducing adherence, while others unintentionally removed or misplaced them, leading to a 40% loss or damage rate in that project. This highlights the need for more discreet, lightweight, and tamper-resistant sensor designs that seamlessly integrate into daily wear. Data collection also faced limitations, as site-specific environmental conditions and seasonal variations may have influenced results. The study’s reliance on a single type of sensor raises concerns about generalizability, particularly in RGB and IR channel sensitivity. Future research should include multisite, long-term studies across diverse lighting conditions and validate models using multiple sensor types to improve accuracy and applicability.

To recapitulate, although the use of LYS Button wearable sensors enabled us to collect continuous personal CL exposure data, factors such as sensor placement variability, individual characteristics, and environmental complexities inherent in nursing homes might affect the generalizability of the developed measurement method. We stress that these issues are common when pioneering new methodologies in real-world settings. By advancing sensor design, expanding case studies, and integrating adaptive lighting solutions, future research can enhance the scalability and effectiveness of personalized circadian lighting assessments. These improvements will support more precise data-driven health care interventions, ultimately contributing to better sleep, cognitive function, and overall well-being in long-term care residents.

### Conclusions and Future Work

Using wearable lighting sensors, we developed and validated calibration and predictive models to evaluate individual lighting exposure in terms of photopic lux, CCT, and circadian lighting levels. Controlled laboratory studies and on-site data collection were conducted to calibrate sensor values, as follows: To adjust the raw data from the LYS Button and improve its accuracy, we developed a simple regression calibration using key sensor outputs—CCT (CCT in Kelvin), IR channel irradiance, and raw illuminance (lux). The regression analysis generated coefficients that reflect each parameter’s contribution to estimating true photopic lux, with the IR measurement showing a particularly strong influence likely due to its sensitivity to daylight variations. Overall, the calibration model, with an adjusted *R*² of 0.858 and with reasonable MAE, shows that although some deviations remain (especially under low light conditions), it reliably aligns the sensor’s outputs with the measured lux values in general indoor lighting scenarios.

As with photopic lux calibration, a simple regression model was developed for CCT using the raw outputs (CCT, IR, and lux) from the LYS Button. The model adjusted the raw CCT values by attributing a strong positive weight to the original CCT output and incorporating the effects of IR and lux, which helped account for variations due to daylight influence. With an adjusted *R*² of 0.982 and an MAE of 342, the calibration effectively corrects the sensor outputs to match the true CCT observed in various lighting environments.

Predictive models were then developed to forecast CS levels based on the calibrated lighting factors, with the random forest model outperforming linear regression, achieving an adjusted *R*² (test) of 0.915 and a cross-validation *R*² of 0.857. The IR value was identified as the most influential factor in predicting circadian lighting levels, further reinforcing the reliability of the model. These results demonstrate the robustness of data-driven modeling for CL assessment, providing a highly accurate and scalable method for personal circadian exposure evaluation.

We then implemented wearable sensors and developed models in a bright light therapy project in 2 assisted-living facilities to demonstrate how these models can be used practically. These field applications in 2 assisted-living facilities revealed notable interindividual variations in CL exposure, with room orientation, daily routines, and engagement in facility activities playing key roles. For instance, residents with higher daytime light exposure, particularly from natural sources, demonstrated more stable circadian patterns, whereas those with limited exposure exhibited signs of disrupted rhythms. Similarly, the residents who have increased engagement in everyday routines, and activities, and availability to steady daylight in the south-facing orientation also had relatively high and stable circadian lighting exposure. When assessed individually only, a specific resident with mild dementia, an east-facing room, and reported nighttime wandering were selected for study on 1 weekend day for 24 hours, and their lighting exposure data were very much in accordance with their behavior and activity patterns within that period. These findings underscore the importance of the lighting assessments at the individual resident level in investigating and optimizing environmental conditions for residents with dementia and related health conditions.

## Supplementary material

10.2196/72338 Multimedia Appendix 1 Datasets used.

10.2196/72338 Multimedia Appendix 2 Model codes.

10.2196/72338 Multimedia Appendix 3 Weekly daytime and nighttime lighting condition variations.

## Abbreviations

CCT: correlated color temperature
CL: circadian light
CL_A_: circadian light adjusted
CS: circadian stimulus
IR: infrared
MAE: mean absolute error
MSE: mean squared error
RGB: red, green, and blue
SPD: spectral power distribution
