# Supplementary material for: Using Wearable Sensors to Measure and Predict Personal Circadian Lighting Exposure in Nursing Home Residents: Model Development and Validation
Source: JMIR Aging. 2025 Sep 11;8:e72338. doi: 10.2196/72338 (PMC12501904; doi:10.2196/72338)
Supplement: Multimedia Appendix 2 [file aging-v8-e72338-s002.docx]

# Load necessary libraries

library(readxl)

library(randomForest)

library(writexl)

# Load the datasets

training <- read_excel("C:/Users/nedag/Desktop/cla/Circadian data modeling.xlsx")

weekly_avg <- read_excel("C:/Users/nedag/Desktop/cla/data3.xlsx")

# Fit a random forest model

output <- randomForest(Cla ~ Kevin_ave + R_ave + G_ave + B_ave + IR_ave + Lux_ave, data = training)

# Make predictions on the weekly_avg dataset

Prediction <- predict(output, weekly_avg)

# Add predictions as a new column in weekly_avg

weekly_avg$Prediction <- Prediction

# Save the updated dataset with predictions to a new Excel file

write_xlsx(weekly_avg, "C:/Users/nedag/Desktop/cla/predictions_10.9.xlsx")
