# Supplementary material for: Using Wearable Sensors to Measure and Predict Personal Circadian Lighting Exposure in Nursing Home Residents: Model Development and Validation
Source: JMIR Aging. 2025 Sep 11;8:e72338. doi: 10.2196/72338 (PMC12501904; doi:10.2196/72338)
Supplement: Multimedia Appendix 3 [file aging-v8-e72338-s003.docx]

Multimedia appendix 3. Weekly daytime and nighttime lighting condition variations


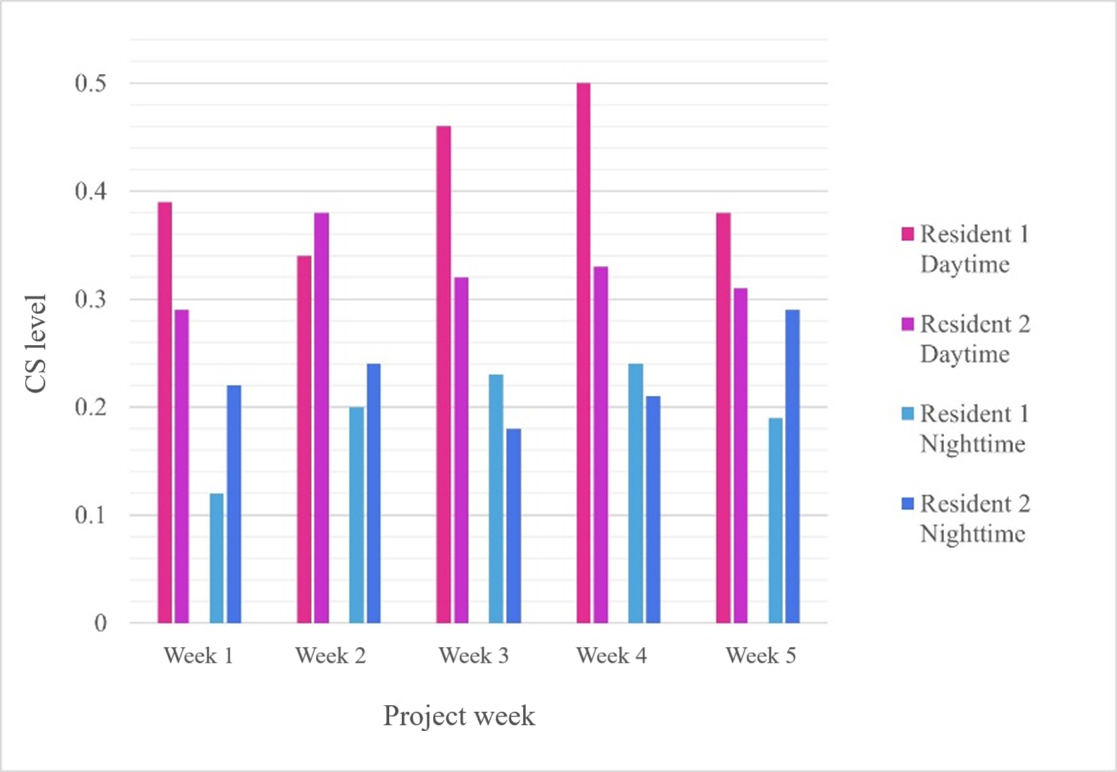


Figure 1. Weekly daytime and nighttime circadian stimulus comparison.


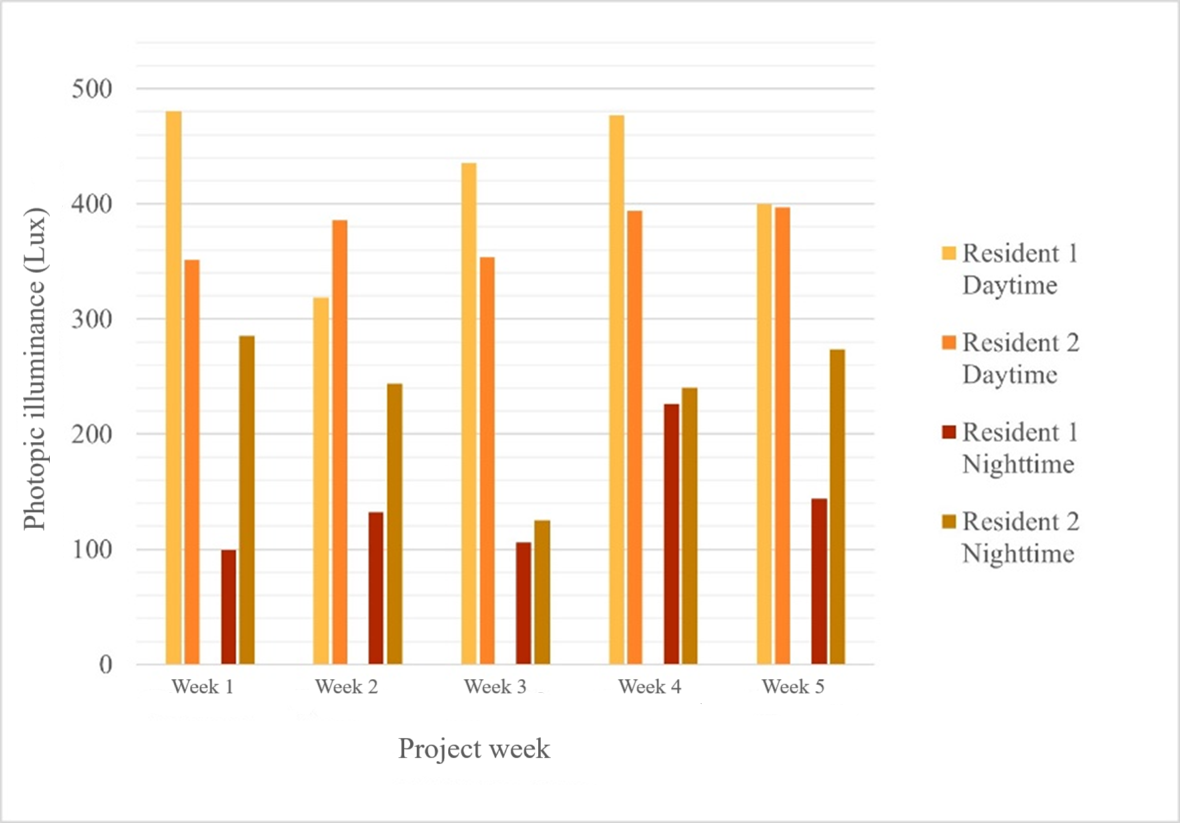


Figure 2. Weekly daytime and nighttime illuminance level comparisons.


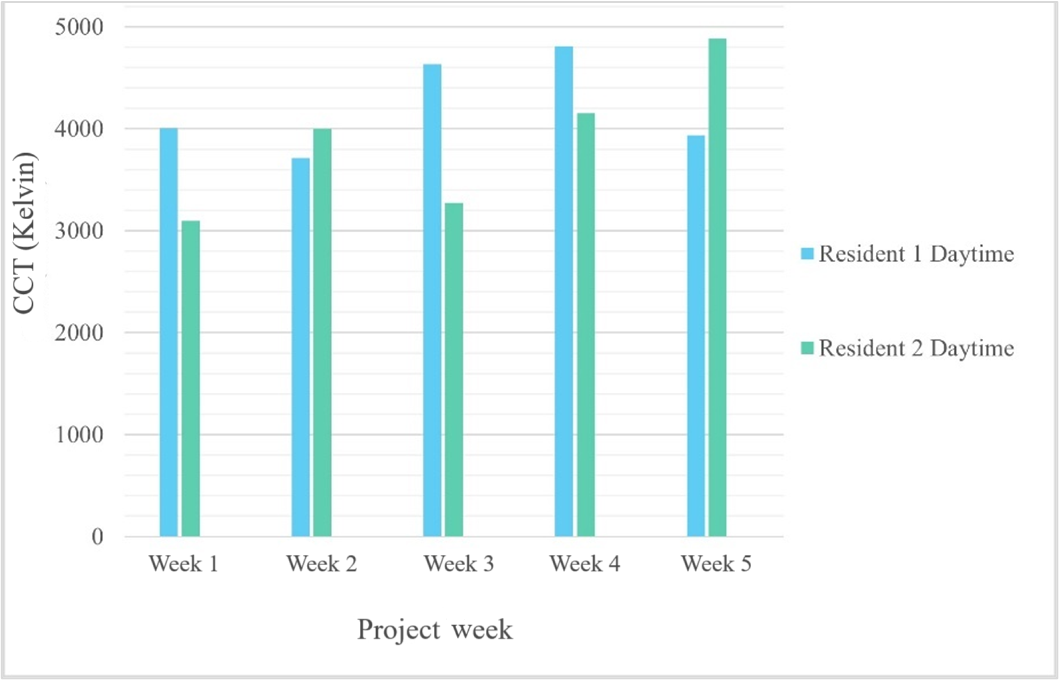


Figure 3. Weekly daytime correlated color temperature inter-variability.
